# A Newly Designed Mobile-Based Computerized Cognitive Addiction Therapy App for the Improvement of Cognition Impairments and Risk Decision Making in Methamphetamine Use Disorder: Randomized Controlled Trial

**DOI:** 10.2196/10292

**Published:** 2018-06-20

**Authors:** Youwei Zhu, Haifeng Jiang, Hang Su, Na Zhong, Runji Li, Xiaotong Li, Tianzhen Chen, Haoye Tan, Jiang Du, Ding Xu, Huan Yan, Dawen Xu, Min Zhao

**Affiliations:** ^1^ Shanghai Mental Health Center Shanghai Jiao Tong University School of Medicine Shanghai China; ^2^ Shanghai South West Weiyu Middle School Shanghai China; ^3^ Shanghai Bureau of Drug Rehabilitation Administration Shanghai China; ^4^ Shanghai Qingdong Compulsory Drug Dependence Rehablitation Center Shanghai China; ^5^ Shanghai Key Laboratory of Psychotic Disorders Shanghai China

**Keywords:** methamphetamine, methamphetamine use disorder, cognitive function, impulse control, risk decision making, attention bias

## Abstract

**Background:**

Cognitive rehabilitation therapy has been found to improve cognitive deficits and impulse control problems in methamphetamine use disorder (MUD). However, there is limited research regarding this therapy’s feasibility when using mobile-based health technologies in supporting recovery from MUD in China.

**Objective:**

The main aim of this study was to test whether 4 weeks of a newly designed computerized cognitive addiction therapy (CCAT) app can improve cognitive impairments, eliminate drug-related attention bias, and attenuate risk decision-making behaviors in participants with MUD.

**Methods:**

Forty MUD participants were assigned randomly to either the CCAT group (n=20), who received 4 weeks of CCAT plus regular detoxification treatment as usual, or the control group (n=20), who only received the regular detoxification treatment as usual, in drug rehabilitation centers in Shanghai. The CCAT was designed by combine methamphetamine use-related picture stimuli with cognitive training with the aim of improving cognitive function and eliminating drug-related attention bias. The CogState Battery, Delay Discounting Task (DDT), Iowa Gambling Task (IGT), and Balloon Analog Risk Task (BART) were administered face-to-face to all participants before and after CCAT interventions.

**Results:**

Forty male patients were recruited. The mean age was 32.70 (SD 5.27) years in the CCAT group and mean 35.05 (SD 8.02) years in the control group. Compared to the control group, CCAT improved working memory in the CCAT group (*P*=.01). Group×time interactions were observed among DDT, IGT, and BART tasks, with rates of discounting delayed rewards, IGT, and BART scores (*P*<.001) being reduced among those who received CCAT, whereas no changes were found in the control group.

**Conclusions:**

The newly designed CCAT can help to improve cognitive impairment and impulsive control in MUD. Further study is needed to understand the underlying brain mechanisms of the cognitive therapy.

**Trial Registration:**

ClinicalTrials.gov NCT03318081; https://clinicaltrials.gov/ct2/show/NCT03318081 (Archived by WebCite at https://clinicaltrials.gov/ct2/show/NCT03318081)

## Introduction

Amphetamine-type stimulants are the second most widely abused illicit drugs worldwide, with methamphetamine being one of the most abused amphetamine-type stimulant drugs, especially in East and Southeast Asia and parts of North America and Europe [1]. Methamphetamine abuse has caused major public health consequences all over the world. Chronic methamphetamine use has been associated with abnormalities in brain function and metabolism [2,3], leading to many negative consequences, including cognitive impairments, high impulsivity, and poor psychological well-being [4].

Cognitive impairments and high impulsivity could lead to a paradox situation in which individuals often desperately continue to consume methamphetamine despite being fully aware of the negative consequences.

According to the dual-systems perspective of addiction, two unbalanced information processing mechanisms underlying methamphetamine use disorder (MUD) patients’ behaviors might address this paradox situation: automatic and reflective processes. Automatic processes, which are overactivated in many substance use disorder (SUD) patients, are fast and automatic impulsive processes, often operating at early stages of response selection when facing high-risk situations [5]. A common feature of the sensitized automatic process is drug-related cognitive bias [6]. The reflective process, which is related to an individual’s cognitive control function, is a considerably slower and relatively controlled process [7]. With continuous drug use, damaged cognitive functions, such as attention control, working memory, and response inhibition, might have a negative effect on this process. The sensitized automatic impulsive process and overslowed reflective process further deteriorates this paradox problem.

Studies have provided evidence that clinical neuropsychology-based rehabilitation techniques focused on cognitive function training and cognitive bias might ideally address this challenge. Computer-based cognitive rehabilitation therapies are one of these promising interventions and have shown beneficial effects for these cognitive deficits across several clinical groups, including schizophrenia [8], brain injury [9], and SUDs [10]. Cognitive bias modification is another computerized treatment technique that targets the sensitized automatic impulsive process. Previous evidence has shown that drug-related attention bias can be retrained, along with favorable short-term effects in reducing substance abuse [11].

Moreover, new technologies can notably enhance the efficacy of health care services. Currently, Web-based and mobile-based health (mHealth) services have been showing promising effects and flexibility in these field [12,13], including addiction treatments [14]. Importantly, these innovative technologies could also better enable service providers in collecting patient behavior data [15] and delivering appropriate interventions [16]. These technologies might help with reaching illicit drug users who were afraid of being stigmatized or monitored by detoxification service providers [17].

However, to the best of our knowledge, no interventions have addressed both cognitive deficits and cognitive bias among MUD patients. Considering both processes are impaired in MUD patients [18], interventions to address these two aspects may be more effective than single approaches [19]. Moreover, there are few published studies that have addressed the efficacy of mHealth technology in delivering cognitive addiction therapies. Therefore, we designed a mobile-based program called computerized cognition addiction therapy (CCAT) by combining cognitive training and cognitive bias modification. Furthermore, studies have shown that enhanced drug-related choice can be demonstrated even for pictorial stimuli [20], with simple passive pictures inducing strong cognitive biases while active pictures presenting drug use-related context induce a stronger urge for drugs [21]. Methamphetamine-related pictures were integrated into the programs, aiming to address both cognitive impairment and cognitive bias in MUD patients. We hypothesized that the CCAT would have beneficial effects in improving cognition impairment, attention bias, and risk decision-making behaviors in MUD patients.

## Methods

### Experimental Design

This study was a randomized, single-blind controlled clinical trial and it has been registered at ClinicalTrials.gov (ID: NCT03318081). All participants were instructed to be treated by computerized cognitive rehabilitation therapies or treatments as usual. All outcome measures were assessed by blinded researchers. The study protocol was approved by the institutional review board at the Shanghai Mental Health Center. All procedures followed were in accordance with the ethical standards of the Norwegian National Committee for Research Ethics in the Social Sciences and the Humanities and with the Helsinki Declaration of 1975, as revised in 2000.

### Mobile-Based Computerized Cognitive Addiction Therapy App

The mobile-based CCAT app was designed to help MUD patients overcome their cognitive deficits, increased control of impulse problems, and enable them to better control methamphetamine-related attentional bias. The CCAT app contains four cognitive training tasks, including two working memory training tasks and two methamphetamine-related attention bias control training tasks. To date, this app is the first mobile app designed for people with MUD in China.

#### Methamphetamine Attention Bias Modification

**Figure 1:**
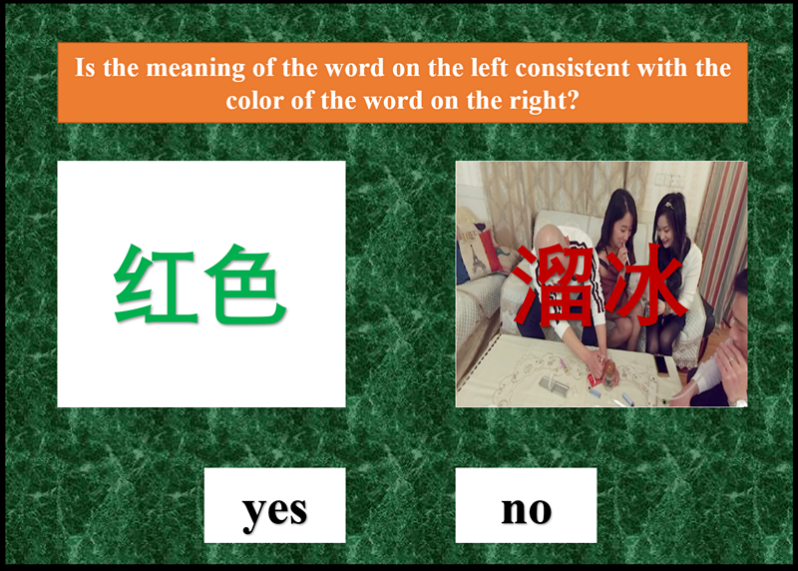
Methamphetamine-related attention bias modification task. Patients were asked to decide whether the meaning of the word in the left box was consistent with the color of the word on the right. The Chinese word printed in green on the left means “red,” whereas the phrase presented on the right means “smoking methamphetamine.”.

Research has shown that attentional bias can be retrained along with good effects in reducing substance use [22]. This training task was based on tasks described by Cox et al [23] and Hester et al [24]. In this newly developed task, methamphetamine-related pictures and words were added, patients were trained to judge whether the meaning of the word in the left box was consistent with the color of the word on the right, while ignoring the interference of methamphetamine pictures and words as quickly as possible (Figure 1).

Each training session began with 20 practice trials to help participants get familiar with the training, and methamphetamine cues were not presented during practice. During the real training, methamphetamine-related words and pictures were shown in the right rectangle. Methamphetamine-related words were the most frequently nominated words among Chinese MUD patients. Methamphetamine-related pictures were drawn from the Internet and adjusted using Adobe Photoshop CS6 (Adobe Systems Incorporated, San Jose, CA, USA) for picture size, exposure, brightness, and contrast. Every session lasted approximately 8 minutes. An incorrect response resulted in a red cross, whereas a correct response resulted in a green checkmark. The colors of the words were limited to red, yellow, blue, and green. Accuracy rates were shown at the end of the training session.

#### Methamphetamine Attention Control Training

**Figure 2:**
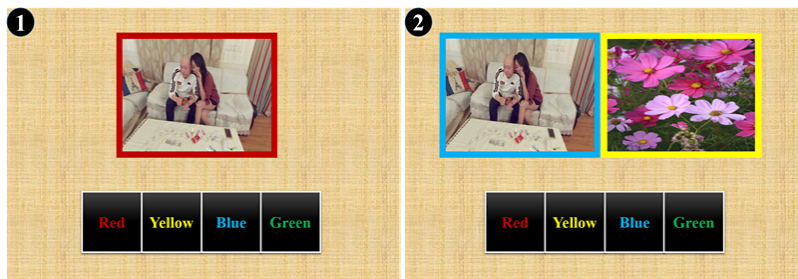
Methamphetamine-related attention control training. In situation 1, the border of the methamphetamine-related image was red, and the patients needed to push the “red” button. In situation 2, the border of the neutral picture was yellow, and the patients needed to push the “yellow” button as quickly as possible.

This training task was derived from the alcohol attention control training project of Fadardi and Cox [25]. Previous studies have shown its efficacy in increasing control over substance-related distraction and reducing substance-related attention bias among SUD patients [26,27]. During the training, when there was only one methamphetamine-related picture, patients needed to determine the border color of the picture. However, if there were two pictures (one was methamphetamine-related and the other was a neutral picture), patients were to ignore the influence of the methamphetamine-related picture and push the button representative of the border color of the neutral picture. Each training task contained 240 trials. Accuracy rates were shown at the end of the training session (Figure 2).

**Figure 3:**
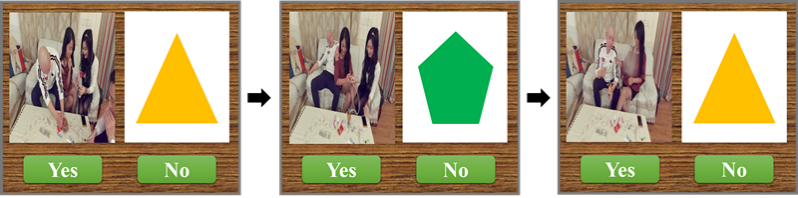
Methamphetamine-related working memory training task (N-back task). The previous Figure 2 is an example of 2-back task training. Patients in the CCAT group were asked to decide the whether the figure (both shape and color) on the right was consistent with the figure showing the previous two pictures while ignoring the methamphetamine-related picture on the left.

#### Working Memory Training (N-Back Task)

This task originated in the N-back task because N-back task-based working memory training has already shown good results in SUD [28] patients. During N-back tasks, participants were required to respond to a specified target, such as letters, figures, or numbers, which appeared on the screen consecutively and were the same as the previous N-back. This part of training was based on a modified version of the N-back task ranging from 1-back to 3-back, with methamphetamine-related pictures set as distractions. For the 1-back task, patients were required to decide whether the figure (eg, triangle, circle, or rectangle) was the same as the previous 1-back. For 2-back and 3-back, patients were required to decide when the current figure showed on screen was the same as the previous 2-back or 3-back earlier. During the training session, the figures shown on the right side of the screen were the target stimuli. Methamphetamine-related pictures on the left served as distractions (Figure 3). Patients identified targets by pressing the “yes” or “no” buttons. Training began with the 1-back task, and the training level was upgraded by achieving 90% accuracy twice in succession. The duration of each training session was set at 10 minutes; the accuracy rates were shown at the end of the training session.

#### Spatial Working Memory Training (Memory Matrix Task)

**Figure 4:**
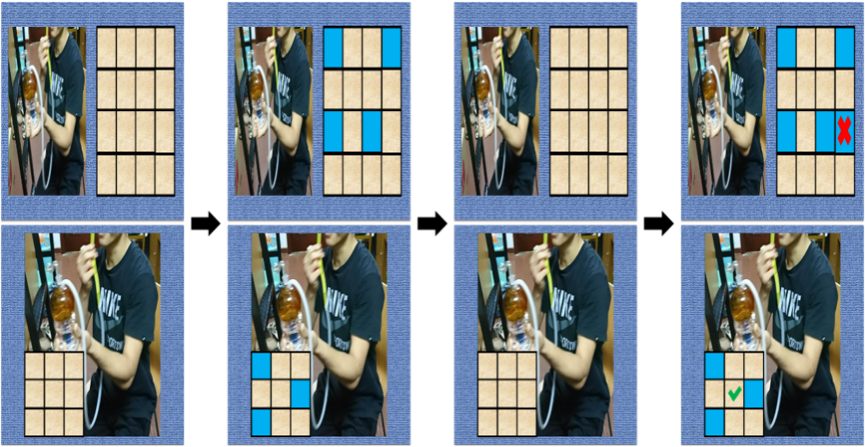
Memory matrix task. A few blue squares were shown for 3 seconds and then they disappeared and returned to the original color. Patients were told to indicate the squares that turned blue that were shown seconds before. An incorrect response resulted in a red cross, whereas a correct response resulted in a green checkmark.

Spatial working memory training could also be improved through computerized visuospatial training [29]. Methamphetamine-related pictures were set as the background (at the bottom of Figure 4) or on the left as a distraction (at the top of Figure 4). During each trial, a rectangle (constituted by many squares) was shown for 1 second. After that, some of the squares turned blue and were shown for 3 seconds. Then they disappeared and returned to its original color. Patients were told to recall these blue squares by pressing the screen for 3 seconds. An incorrect response or failure to respond in 3 seconds resulted in a red cross, whereas a correct response resulted in a green checkmark. Three times were set as the response threshold. Training began with recalling three figures. If the patient successfully recalled or failed to remember the blue squares three times in succession, then the figures to be recalled were increased or decreased accordingly. Accuracy rates were shown at the end of the training session. The duration of each training session was set at 10 minutes.

### Participants

**Figure 5:**
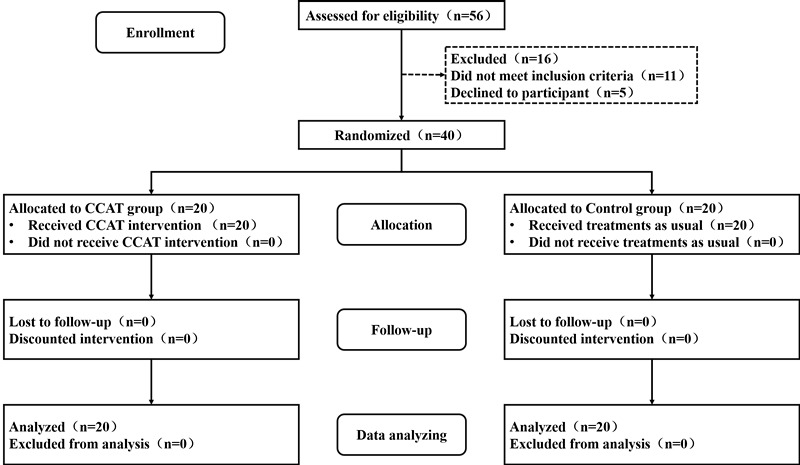
CONSORT flowchart of the study. CCAT: computerized cognitive addiction therapy.

A total of 40 male participants from one compulsory rehabilitation center in Shanghai who met the *Diagnostic and Statistical Manual of Mental Disorders* (Fifth Edition) criteria for moderate or severe MUD were recruited to participate in the study. Inclusion criteria were (1) more than 9 years of education, (2) aged 18 to 49 years, (3) normal vision and audition, (4) receive no detoxification medications during treatment, (5) right handedness, and (6) no current use of methamphetamine or any other substances (except nicotine) for at least 7 days. Exclusion criteria included (1) current medical diseases that required hospitalization or regular monitoring; (2) serious physical or neurological illness that required pharmacological treatment affecting cognitive function; (3) history of major psychiatric disorders such as bipolar disorder, schizophrenia, depression, and disorders of high comorbidity with SUD; (4) neurological diseases such as stroke, seizure, migraine, and head trauma; (5) intelligence quotient of less than 70; and (6) color blindness (see CONSORT flowchart in Figure 5). Written consent forms were obtained from all participants.

### Data Collection and Measurements

#### Demographic and Methamphetamine Use Information

Each participant was interviewed by one trained psychiatrist to collect the individual’s sociodemographic characteristics and methamphetamine use histories.

#### Cognitive Function

Cognitive function was assessed using the Chinese version of the CogState Battery, which has good validity (Cronbach alpha=.8) [30]. The CogState Battery includes five cognitive tasks that assess verbal learning and memory, working memory, spatial working memory, problem solving/error monitoring, and social cognition. The total number of correct responses during the International Shopping List (ISL) task were used to reflect verbal learning and memory ability. Working memory was evaluated through the proportion of correct responses during the two-back task performance. Spatial working memory functions were reflected through the total number of errors in the continuous paired association learning (CPAL) task. The problem-solving/error-monitoring function was assessed by the Groton maze learning task, its assessment index was the same as CPAL during the Groton maze learning task. For social cognition function, the proportion of correct responses of the social emotional cognition task were applied.

#### Iowa Gambling Task

During the Iowa Gambling Task (IGT), participants were asked to choose among four decks of cards (A, B, C, and D) and accumulate as much money as possible by selecting one card at a time. Decks A and B were associated with high immediate wins, but larger future penalties that resulted in a net loss over time (ie, the disadvantageous decks). Decks C and D yielded lower immediate wins but smaller future penalties, such that participants gradually accumulated a profit by choosing these decks (ie, the advantageous decks). There were a total of 150 trials. The outcome was the net score (the number of cards from the disadvantageous decks subtracted from the advantageous decks). A positive score reflected the individual having a tendency to make a good decision [31]. Poor decision making was indicated by a lower IGT score.

#### Balloon Analog Risk Task

The Balloon Analog Risk Task (BART) was a measurement of the individual’s risk-taking behavior [32]. On each single trial, an uninflated balloon appeared on the screen and pressing the button “1” inflated the balloon, and with each successful trial, the patient received 10 points and the balloon became considerably larger. Because the balloon had the possibility of explosion on each inflation, participants needed to press button “5” to stop inflating the balloon and obtain the benefit in time. In total, there were 100 trials. Before the test started, participants were told to obtain as high a score as possible, and total scores were presented on the lower-right corner of the monitor. BART scores (total number of balloon inflations/total number of unexploded balloons) were used to assess impulsive risk-decision making.

#### Delay Discounting Task

In the Delay Discounting Task (DDT), the delayed reward was set at 1000 Chinese yuan (approximately US $158); delay times were 2 days, 1 week, 1 month, 3 months, 6 months, and 1 year. The beginning immediate reward was 500 Chinese yuan (approximately US $15.80). Participants were told to choose the smaller immediate reward over the larger but delayed reward. The larger delayed reward stayed the same, whereas the immediate reward changed from trial to trial according to a decreasing-adjustment algorithm until an indiﬀerence point was recorded (an indifference point means the participant changed choice between immediate and delayed rewards). The hyperbolic decay model was used to calculate the discounting rate (k) to reflect the individual’s risky-decision making function [33]. A higher k meant the participant had much higher impulsiveness.

#### Methamphetamine Stroop Task

A Chinese version of the methamphetamine addiction Stroop task was applied to measure the methamphetamine-related attentional bias. The words used involved eight methamphetamine-related words and neutral words. Each of the 16 words was presented eight times in four different colors (red, green, yellow, and blue). Every word was shown on the screen for 3000 milliseconds. Participants were asked to ignore the meaning of the words by pressing the buttons corresponding to the color of the word presented as quickly as possible. Stimuli were presented in a pseudorandomized, nonstationary probabilistic sequence. Reaction time and errors rates were recorded. Attention bias was calculated by subtracting the time needed to name the color of the neutral words from the time taken to complete the methamphetamine-related words.

Cognitive tasks were programmed by E-prime 2.0 (Psychology Software Tools, Inc, Sharpsburg, PA, USA).

#### Procedures

Participants were randomly assigned to the CCAT group or control group by researchers who were not involved in other parts of this research. Pretraining and posttraining assessments were conducted by two other well-trained doctors. During the treatment, patients in the control group only received standard treatment in a compulsory rehabilitation center. Participants included in the CCAT group were also undergoing standard treatment; in addition, the participants received the CCAT training program that lasted for 4 weeks (20 sessions, five times a week, each session lasts approximately 60 minutes).

The CCAT training programs were displayed on an iPad. During each training session, participants in the CCAT group completed each of the four training programs twice. Standard treatment included health education (45 minutes per session, once a week), judicial education (45 minutes per session, once a week), sports activities (60 minutes per day), and vocational training (45 minutes per session, twice a week). Patients in the control group did not receive this type of CCAT training, and they only participated in the assessments at baseline and 4 weeks later.

Importantly, pictures involving drug-related stimuli and cues could still induce the individual’s attention bias and craving for drugs [20]. After every CCAT session, a 5-minute relaxation session was carried out to relieve possible psychological reactions and cravings induced by the methamphetamine-related cues. Relaxation included playing light music and watching pictures with relaxing effects.

### Safety

Safety was assessed at every treatment session with a self-administered CCAT training form by recording spontaneous adverse events such as headaches and dizziness.

### Statistical Analyses

Data were analyzed using SPSS 21.0. Group differences were compared using student’s *t* test or analysis of variance (ANOVA) for continuous variables and a chi-square test for categorical variables. Generalized estimating equations were used to assess the main effects of groups (CCAT group vs control group), time (pretreatment vs posttreatment), and group×time interactions for all cognitive test variables. Statistical significance was set at alpha=.05. Bonferroni’s test was used to resolve significant interactions for post hoc analysis.

## Results

### Demographic and Methamphetamine Use Information

Demographic characteristics and drug use histories are shown in Table 1. There were no differences between the CCAT group and control group in terms of mean age, education, marriage, onset age of first methamphetamine use, abstinence time, duration of methamphetamine use, dose, and frequency.

### Effect of Computerized Cognitive Addiction Therapy on Cognitive Function

**Figure 6:**
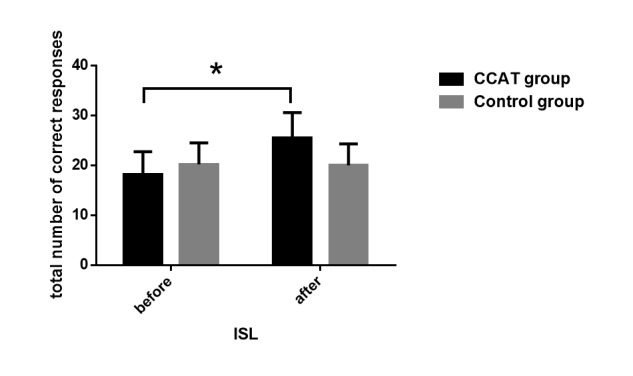
International Shopping List (ISL) scores before and after intervention. Verbal learning and memory function were evaluated by ISL; scores are total number of correct responses. Significant differences between the two groups (P<.001) are marked by the asterisk. CCAT: computerized cognitive addiction therapy.

**Figure 7:**
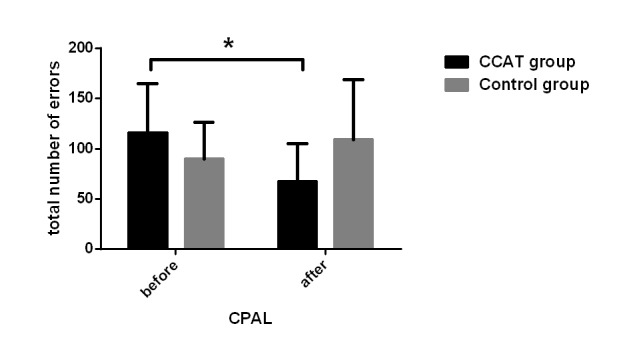
Continuous Paired Association Learning (CPAL) scores before and after intervention. Spatial working memory functions were reflected through the total number of errors in the CPAL. Significant differences between the two groups (P=.01) are marked by the asterisk. CCAT: computerized cognitive addiction therapy.

**Figure 8:**
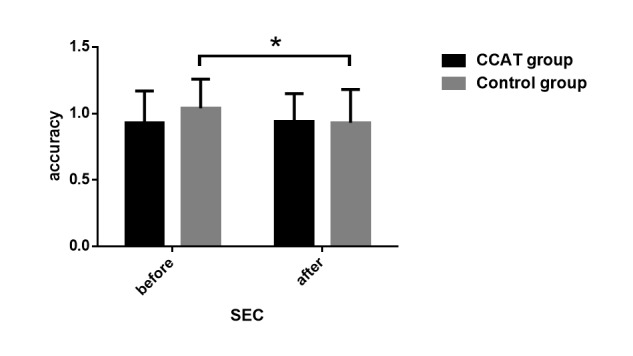
Social emotional cognition (SEC) task scores before and after intervention. Social cognition was evaluated by the SEC task; SEC scores were assessed by accuracy rate (the proportion of correct responses). Changes in SEC scores did not reach significant level in CCAT group (P=.56), whereas the accuracy rate decreased significantly in the control group (P=.02) as reflected by the asterisk. CCAT: computerized cognitive addiction therapy.

The CogState test, ISL, and CPAL scores increased significantly in the CCAT group, whereas the control group patients did not show significant changes. A significant time×group effect (*F*_1,1_=31.78, *P*<.001), group effect (*F*_1,1_=4.53, *P*=.03), and time effect (*F*_1,1_=9.37, *P*<.001) were observed in ISL scores. Group effect (*F*_1,1_=5.95, *P*=.02) and time effect (*F*_1,1_=5.45, *P*=.02) in CPAL scores also reached a significant level. Although the group×time effect (*F*_1,1_=6.68, *P*=.01) was significant for social emotional cognition scores, patients in the control group decreased significantly compared to the CCAT group. Groton maze learning and 2-back task scores did not show a significant change between the groups (Figures 6-8).

### Effect of Computerized Cognitive Addiction Therapy on Impulsive Risk-Decision Making

**Figure 9:**
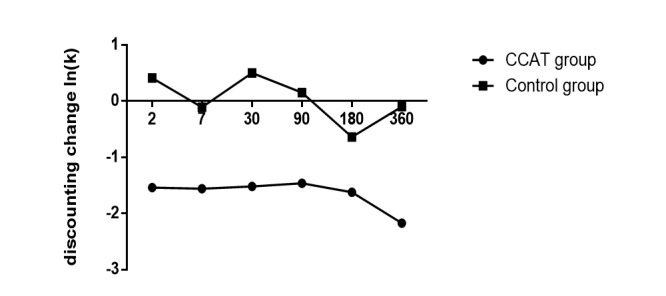
Discounting change ln(k) before and after computerized cognitive addiction therapy (CCAT) training. Change in discounting ln(k) for participants in CCAT and control groups, calculated as posttraining minus pretaining. Negative values indicate a decrease in discounting. The values 2, 7, 30, 90, 180, and 360 were delayed times in the delay discounting task.

**Figure 10:**
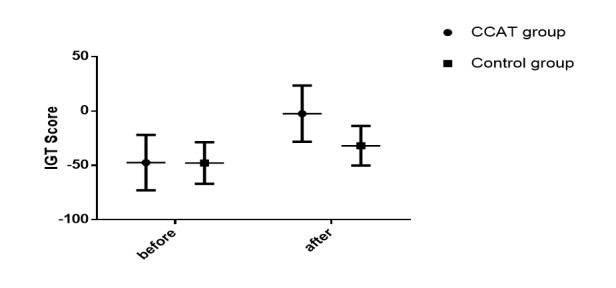
Iowa Gambling Task (IGT) scores after computerized cognitive addiction therapy (CCAT) or control training. The IGT score was calculated through the number of cards from the disadvantageous decks (C and D) subtracted from the advantageous decks (A and B). A positive score reflects the individual had a tendency to make better decisions. The lines are means and the error bars are the standard deviation.

A training effect was observed in the CCAT group for risk decision-making tasks. First, those undergoing CCAT training significantly decreased their discounting rate, whereas there was no significance in the control group. The group×time interaction effect was significant at each of the delayed times (Figure 9). A training effect was also observed with measures of IGT (Table 1 and Figure 10). The treatment×time effect, group effect (*F*_1,1_=4.84, *P*=.03), and time effect (*F*_1,1_=214.60, *P*<.001) were significant (*F*_1,1_=49.07, *P*<.001). In the BART test, a significant group×time interaction (*F*_1,1_=22.75, *P*<.001) and time effect (*F*_1,1_=5.16, *P*=.02) had reached a significant level. Further comparison showed the CCAT group had better performance than the control group after CCAT invention.

### Effect of Computerized Cognitive Addiction Therapy on Attention Bias

There were no significant differences between the two groups in attention bias. The treatment×time effect did not reach a significant level (*F*_1,1_=0.92, *P*=.34). Only a time effect (*F*_1,1_=6.23, *P*=.01) was observed (see Multimedia Appendix 1).

### Safety

No patients reported any discomfort during the whole training session.

**Table 1:** Demographic and drug use characteristics of participants (N=40).

Characteristics		CCAT^a^ group (n=20)	Control group (n=20)	*F*_1,38_	χ^2^_3_	*P*
Age (years), mean (SD)		32.70 (5.27)	35.05 (8.02)	1.200		.28
Education (years), mean (SD)		10.00 (2.43)	9.55 (1.36)	0.525		.47
Age of onset (years), mean (SD)		24.45 (6.54)	25.15 (8.56)	0.084		.77
Abstinence (months), mean (SD)		4.30 (1.17)	4.10 (1.18)	0.224		.64
Duration of methamphetamine use (year), mean (SD)		6.02 (3.72)	7.00 (2.73)	0.891		.35
Dose of methamphetamine use (g/day), mean (SD)		0.60 (0.31)	0.66 (0.39)	0.294		.59
**Frequency of methamphetamine use, n (%)**					2.4	.56
	Everyday	14 (70%)	10 (50%)			
	3-5 times a week	4 (20%)	8 (40%)			
	Once a week	1 (5%)	1 (5%)			
	1-3 times a month	1 (5%)	1 (5%)			

^a^CCAT: computerized cognitive addiction therapy.

## Discussion

### Principal Findings

To the best of our knowledge, this report describes the first pilot study that combined cognitive training and cognitive bias modifications adding methamphetamine-related stimulants in MUD treatment programs. As expected, compared to the control group, patients engaged in the CCAT group had better cognitive performance after 4 weeks of training, which coincided with changes in impulsive risk decision-making tasks measured by DDT, IGT, and BART. These results supported our hypotheses that 4-week CCAT training would have beneficial effects for improving cognition impairment and risk decision-making levels in MUD patients.

Previous studies have shown that computerized cognitive training showed promising treatment effects and even brain plasticity in SUD patients using such stimulants as cocaine, methamphetamine, alcohol, and nicotine [34]. However, these studies only used cognitive training, such as working memory training, or cognitive bias modification training. Although this research has provided primary evidence for combining general cognitive training, attentional bias retraining may further enhance the treatment prognosis.

One notable finding is that, along with cognitive function improvement, patients in the CCAT group also showed better performance of impulsive control tasks, which is in line with previous studies, indicating that good working memory functioning could allow for better decision making in SUD patients [35]. These studies have already indicated the underlying relationship between cognitive training and impulsive control enhancement, either by cognitive tasks or self-report measures [36,37]. In this study, our results not only revealed a similar trend but also for cognitive tasks, such as DDT and IGT. Importantly, previous study either included people with stimulant dependence, including cocaine and methamphetamine, or self-report measures. However, in our study, we only recruited patients with moderate to severe MUD, and these patients had better performance on cognitive tasks. Thus, although our findings are small and preliminary, our pilot study provided further evidence that cognitive training can affect impulsive control rehabilitation in MUD patients.

Unexpectedly, CCAT training did not show improvement in social cognition. However, preevaluation and postevaluations of social emotional cognition tasks revealed that patients in the control group showed a trend of deterioration. Our previous study found that social cognition also showed dysfunction in individuals with MUD [38]. Other studies have proved that enhancing working memory capacity could better help patients dealing with negative social emotional events [39]. Therefore, our research may indicate that 4 weeks of CCAT was not long enough to facilitate the recovery of social cognition and may serve as protective factors.

Although studies have indicated that substance-related attention bias can be retrained with favorable short-term effects and clinical effectiveness [40], in this research, attention bias did not show significant changes after CCAT training. Other studies also suggested home environments and mobile technology may promote robust reductions in bias and clinical effectiveness [41]. Our previous study on methamphetamine-related attention bias also showed that, although there was no significant difference in behavioral performance between MUD and healthy controls, increased P300 amplitudes by methamphetamine-related words were observed among MUD patients compared to healthy controls [42]. However, this report described a small pilot study, and well-powered clinical trials combined with event-related potentials are required to obtain a more conclusive answer on the potential clinical effectiveness of attention bias modification.

From a clinical standpoint, there is still no official approved medical treatment for MUD patients, making finding new treatment approaches for MUD patients of great importance. Preserved cognitive function and decision making were key factors underlying patients’ long-term prognoses [43]. These were consistent with evidence demonstrating that working memory and its interaction with impulsive risk-decision making could predict levels of substance use during treatment [44]. Therefore, strengthening these abilities during treatment might facilitate the individual’s resilience and reduce relapse. Our research did show that 4-week CCAT training could enhance both patients’ working memory ability and risk decision-making level. However, whether CCAT training can be implemented as conventional treatment in MUD patients and its mechanism remains a fertile area for further research.

### Limitations

This study has several limitations. First, we employed a relatively small sample (n=20 in each group). We only recruited male patients in our study, which limited further highlighting of the difference in clinical efficacy of cognitive training. Given various constraints, we were unable to increase the number of participants to be recruited; however, considering our baseline data showed a normal distribution and homogeneity of variance, which could still increase the validity of our findings. The second limitation was that in order to make each task more engaging and relevant, we added methamphetamine-related pictures during training. However, we did not have enough sufficient trials to explore stimulus effects in these patients. To keep the training as effective as possible, we invited these patients to rate the pictures (valance, arousal, dominance, and craving) and only used pictures that exceeded five points. Third, given the increased risk of induced craving during CCAT training, we were careful about the side effects of CCAT. Relaxation was conducted at the end of each training session and no patients reported discomfort after the invention. Another limitation was the lack of data assessing the individual’s motivation to participate in CCAT training. Because motivation level is an important moderator of the effectiveness of cognitive training and adherence [45], our future research would combine varieties of motivational approaches as a plug-in with a newly upgraded CCAT app to collect data on patients’ motivational levels.

### Conclusions

The results from our study support the fundamental dual process theory underlying cognitive based treatments for MUD individuals. Four weeks (20 sessions) of CCAT training could both better facilitate cognitive function rehabilitation and reduce impulsivity-related decision making in participants. Future studies will focus on functional magnetic resonance imaging and electroencephalograms to find the underlying mechanisms of CCAT.

## Appendix

Multimedia Appendix 1 IGT and BART score and attention bias scores before and after CCAT intervention.

Multimedia Appendix 2 CONSORT‐EHEALTH checklist (V 1.6.1).

## Abbreviations

BART: Balloon Analog Risk Task
CCAT: computerized cognitive addiction therapy
DDT: Delay Discounting Task
IGT: Iowa Gambling Task
ISL: International Shopping List
MUD: methamphetamine use disorder
SUD: substance use disorder
