# Supplementary material for: A Newly Designed Mobile-Based Computerized Cognitive Addiction Therapy App for the Improvement of Cognition Impairments and Risk Decision Making in Methamphetamine Use Disorder: Randomized Controlled Trial
Source: JMIR Mhealth Uhealth. 2018 Jun 20;6(6):e10292. doi: 10.2196/10292 (PMC6031898; doi:10.2196/10292)
Supplement: Multimedia Appendix 1 [file mhealth_v6i6e10292_app1.pdf]

IGT and BART score and attention bias scores before and after CCAT intervention

Task	CCAT group（M ± SD）		Control group（M ± SD）		Time	Group	Time*Group
	Before	After	Before	After	（F，p）	（F，p）	（F，p）
IGT	-47.45 ± 25.48	-2.40±25.83	-47.80±19.14	-31.90±18.17	4.84,0.03*	214.60,0.00*	49.07, 0.00*
BART	10.68 ± 5.08	6.95 ± 2.31	9.34 ± 3.47	8.03 ± 2.08	5.16, 0.02*	0.02, 0.90	22.75, 0.00*
Attention bias	5.19 ± 2.52	3.08 ± 1.49	5.17 ± 2.85	4.23 ± 2.97	6.23, 0.01*	0.77, 0.38	0.92, 0.34

* $p < 0.05$, M = mean, SD = standard deviation
